# Exercise-induced modulation of myokine irisin in bone and cartilage tissue—Positive effects on osteoarthritis: A narrative review

**DOI:** 10.3389/fnagi.2022.934406

**Published:** 2022-08-19

**Authors:** Ke Ning, Zhuo Wang, Xin-an Zhang

**Affiliations:** College of Kinesiology, Shenyang Sport University, Shenyang, China

**Keywords:** osteoarthritis, bone mineral density, bone metabolism, cartilage, cartilage metabolism, exercise, myokines, irisin

## Abstract

Osteoarthritis is a chronic degenerative musculoskeletal disease characterized by pathological changes in joint structures along with the incidence of which increases with age. Exercise is recommended for all clinical treatment guidelines of osteoarthritis, but the exact molecular mechanisms are still unknown. Irisin is a newly discovered myokine released mainly by skeletal muscle in recent years—a biologically active protein capable of being released into the bloodstream as an endocrine factor, the synthesis and secretion of which is specifically induced by exercise-induced muscle contraction. Although the discovery of irisin is relatively recent, its role in affecting bone density and cartilage homeostasis has been reported. Here, we review the production and structural characteristics of irisin and discuss the effects of the different types of exercise involved in the current study on irisin and the role of irisin in anti-aging. In addition, the role of irisin in the regulation of bone mineral density, bone metabolism, and its role in chondrocyte homeostasis and metabolism is reviewed. A series of studies on irisin have provided new insights into the mechanisms of exercise training in improving bone density, resisting cartilage degeneration, and maintaining the overall environmental homeostasis of the joint. These studies further contribute to the understanding of the role of exercise in the fight against osteoarthritis and will provide an important reference and aid in the development of the field of osteoarthritis prevention and treatment.

## Introduction

Osteoarthritis (OA) is the most common musculoskeletal disease. According to incomplete statistics, OA affects ~240 million people worldwide, representing ~3.8% of the total population worldwide. The prevalence of osteoarthritis is significantly higher in older adults, and that the prevalence in adults older than 65 years is accounting for more than 1/3 of the total population (Hawker, 2019). Its prevalence is expected to continue to rise significantly in the future, along with the increasing aging of the population. Osteoarthritis has become a global public health problem (Kloppenburg and Berenbaum, 2020). It is characterized clinically by loss of articular cartilage and subchondral bone changes (Donell, 2019), causing outcomes, including joint space narrowing, joint pain, stiffness, deformity, and even disability (Suri et al., 2012; Kraus et al., 2015; Katz et al., 2021). OA greatly affects the quality of life of patients and places a considerable medical burden on individuals and society (Sharif et al., 2015), so the medical burden on individuals and society is considerable (Sharif et al., 2015).

Exercise is considered to be a key factor in treating osteoarthritis (Nelson et al., 2014) and is central to a non-pharmacological treatment (Regnaux et al., 2015). Physical activity is based on the skeletal muscle activity (Rannou and Poiraudeau, 2010). Exercise therapy (Fransen and McConnell, 2009) can be effective in reducing the pain associated with osteoarthritis, improving physical function, and (Penninx et al., 2001; Latham and Liu, 2010) significantly reducing the risk of disability in osteoarthritis (Alghamdi et al., 2004). Notably, age does not appear to affect the benefits of exercise training (Nelson et al., 2014), and the improvements in joint function following training are similar in older and younger people. Despite the decline in dependency among the elderly, exercise has been generally shown to delay skeletal sarcopenia and osteopenia and to reduce the risk of diseases, such as mechanical arthritis of the knee and hip, that accompany the process of muscle loss (Bains, 2020). Therefore, exercise deserves further attention as a relatively safe treatment method (Bricca et al., 2020), but the mechanisms that exercise modulating osteoarthritis are, as yet, unclear.

**Figure 1:**
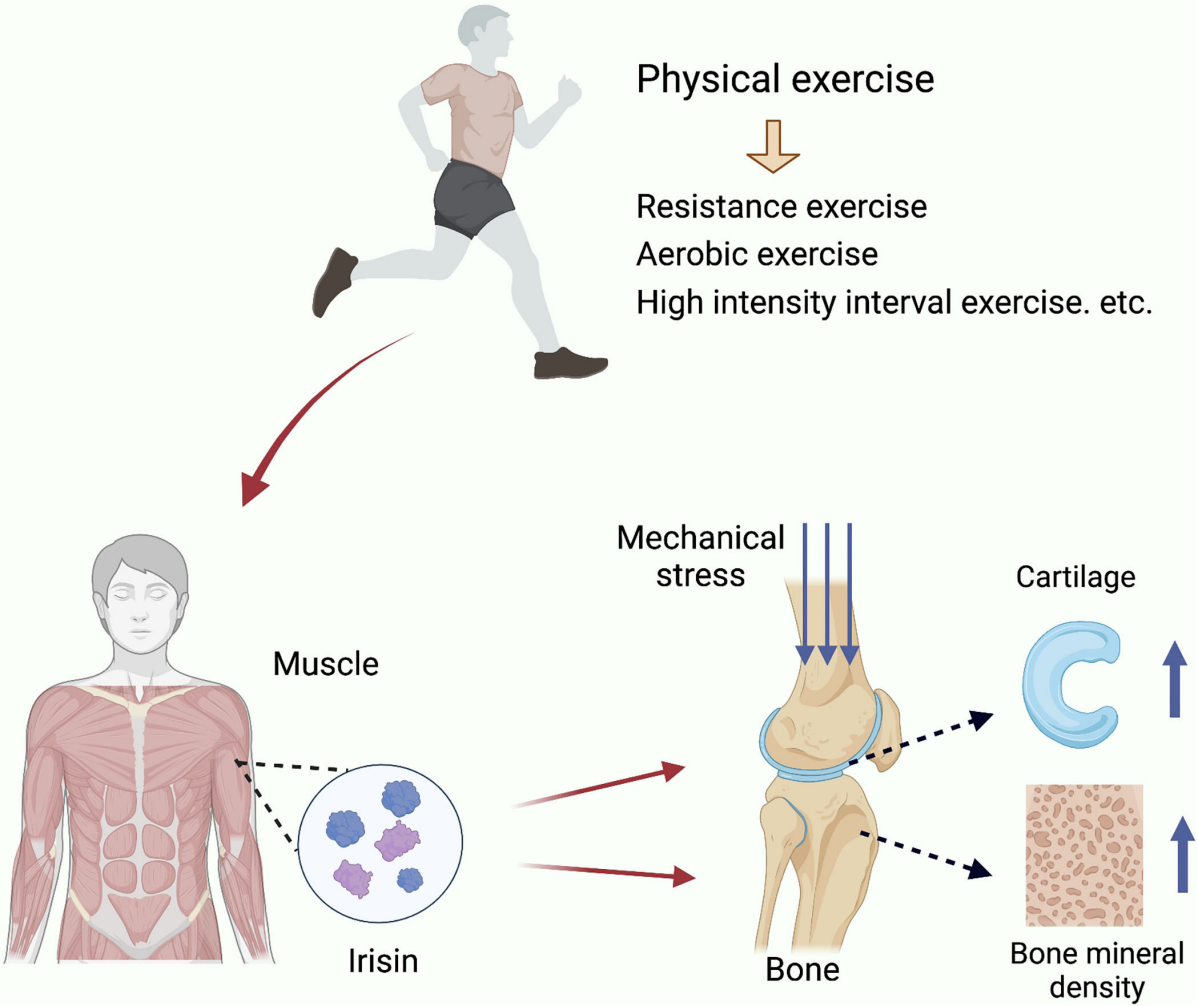
Exercise inducted effects of Iris and its regulation in OA. Irisin is secreted by skeletal muscle in response to exercise stimulation and increases bone density, enhances the mechanical support of cartilage by subchondral bone, improves cartilage tissue, and promotes chondrocyte proliferation.

Current research studies have found that exercise can act as a secretory organ by stimulating the skeletal muscle system *in vivo* (Pratesi et al., 2013), causing the release of myokines (Pedersen, 2011; So et al., 2014) and providing new ideas to explain the beneficial effects of exercise in the treatment of osteoarthritis. Myokines, a class of cytokines or peptides synthesized and secreted by muscle fibers during muscle contraction (Pedersen et al., 2003), act on the muscle itself and other organs in an autocrine, paracrine, and endocrine form (Pedersen and Febbraio, 2012). Myokines are involved in regulating metabolic processes and achieving coordination between skeletal muscle and organs (Trayhurn et al., 2011; Pedersen and Hojman, 2012; Severinsen and Pedersen, 2020). In 2012, a new myokine was discovered and named irisin (Bostrom et al., 2012). It is produced by cleavage of fibronectin type III domain containing protein 5 (FNDC5) and has been shown to be induced by exercise (Wrann et al., 2013). Since its discovery, irisin has been reported to regulate a variety of metabolic disorders (Polyzos et al., 2018) acting in a variety of tissues, including bone. Exercise-induced irisin plays a potentially important role in the prevention and resistance to the progression of osteoarthritis (Figure 1). In the past, many studies have focused on the role of irisin in the regulation of bone metabolic homeostasis. In recent years, there has been an increasing interest in research articles on the role of irisin in cartilage and the extracellular matrix of cartilage, some of which have been published. However, the role of irisin in the development of osteoarthritis has not been systematically reviewed. An overview of the regulatory role of irisin in bone and cartilage tissues would help to gain insight into the development of osteoarthritis and explain the molecular mechanisms of exercise against osteoarthritis. In this article, we review the studies on the exercise-induced regulation of bone density and articular cartilage by the myokine irisin, and discuss the implications of irisin in the treatment of osteoarthritis.

## Generation and structure of irisin

Irisin is a newly identified myokine that is released into circulation primarily from skeletal muscle (Bostrom et al., 2012). Since the concept of myokines was introduced in 2003 (Pedersen and Febbraio, 2012), more than 300 myokines have been identified in the last 20 years, such as interleukin-6 (IL-6), interleukin-15 (IL-15), fibroblast growth factor 21 (FGF21), myostatin, among others, which also includes irisin. These muscle factors have been shown to be involved in adipose tissue, liver, bone (Trayhurn et al., 2011; Zymbal et al., 2019), central nervous system (Voss et al., 2019; Lee et al., 2021), and immune system (Flori et al., 2021). They play a role in driving browning of white fat (Lee et al., 2014), insulin-sensitive states (Lee et al., 2015), optimizing whole-body energy metabolism, preventing metabolic diseases, and playing a protective role in low-level inflammation on a systemic scale (Tanabe et al., 2017; Flori et al., 2021).

It has been shown that irisin is a cleaved and secreted fragment of fibronectin type III structural domain protein 5 (FNDC5). FNDC5 is a transmembrane protein consisting of 209–212 amino acid residues with a typical fibronectin III structural domain. The c-terminal fragment is located in the cytoplasm and n-terminal portion is located outside the cell that can be cleaved by protein hydrolysis to produce a hormone. The n-terminal fragment is located in the cytoplasm, while its extracellular portion can be cleaved by protein hydrolysis to produce a hormone substance known as irisin (Bostrom et al., 2012; Flori et al., 2021). Irisin consists of 112 amino acid residues that contain two glycosylation sites. Irisin is released into the circulation after glycosylation to exert hormonal effects. Its amino acid sequence is highly conserved in mammals, demonstrating its stable function across species. Irisin, which usually occurs as a homodimer (Schumacher et al., 2013), has been shown to be expressed in almost all tissues and organs of eukaryotes and is particularly highly expressed in skeletal muscle and skeletal muscle-rich tissues.

Most studies suggest that exercise induces the expression and secretion of irisin in muscle tissue. Exercise stimulation promotes the production of irisin by FNDC5 cleavage (Bostrom et al., 2012; Huh, 2018). Several studies have shown that exercise can activate the mitogen-activated protein kinase (MAPK) pathway and increase the expression of peroxisome proliferator-activated receptor gamma coactivator 1α (PGC-1α) transcript levels (Akimoto et al., 2005; Guo et al., 2021). PGC-1α is a transcriptional coactivator that is thought to play a key role in lipid and metabolic regulation (Cheng et al., 2018), that directly regulates the expression of FNDC5-irisin (Bostrom et al., 2012). According to a study in 2018, PGC-1α expression was elevated in muscle cells during simulated exercise with the transcription factor CREB, and overexpression of PGC-1α in complex with CREB-activated FNDC5 transcription in C2C12 cells (Yang et al., 2018). However, Pekkala et al. (2013) found that changes in FNDC5 expression were not consistent with serum irisin, which predicts the existence of other pathways than transcription for irisin release in muscle. At present, the complete mechanisms of irisin production are not clear and need further study.

## Effects of different types of exercise on irisin

Exercise induces irisin expression, and most studies concluded that moderate intensity and resistance exercise significantly increased irisin levels (Liu et al., 2021). However, different types of exercise showed greatly different effects on the induction of irisin (Supplementary Table 1). In a recent study, Tavassoli et al. designed a resistance exercise training (RET) experiment for rats on a high-fat diet. After 12 weeks of training, serum levels of total cholesterol (TG) and triglycerides (TC) decreased and serum concentrations of irisin increased in the rats participating in the RET. Tavassoli et al. (2022) hypothesized that the increased serum levels of irisin in rats were related to muscle contraction during training. In a 2019 mouse experiment, exercise-induced irisin exerted an anti-oxidative stress effect *via* Nrf2 and improved smoking-induced emphysema (Kubo et al., 2019). Li et al. also recently demonstrated that different types of exercise, including aerobic, resistance and vibration exercise, and skeletal muscle electrical stimulation all upregulated irisin/FNDC5 expression in mouse myocardium, which resulted in promoting mitochondrial phagocytosis, enhancing antioxidant function, and thus improving cardiac function with having a more significant effect of resistance exercise (Li H. et al., 2021). There is a prominent role of resistance exercise in increasing serum irisin concentrations (Tsuchiya et al., 2015; Cosio et al., 2021). In addition, a study in 2014 compared in detail serum irisin levels at different stages of exercise in subjects with different levels of training in two different forms of exercise, cycling and sprinting (Huh et al., 2014). The researchers found that irisin expression was independent of the type of acute exercise and the training status of the subjects, but that running caused a longer duration of irisin elevation compared to cycling. The difference in the duration of irisin upregulation may be due to the higher rate of fat oxidation in running compared to cycling at the same relative intensity (Capostagno and Bosch, 2010). In a recent study, Colpitts et al. (2022) reported that during aerobic exercise at 35 min, high-intensity interval (HIIT) training triggered a higher peak irisin response in the serum of healthy adolescents compared to moderate continuous intensity (MCI) exercise. HIIT triggered a higher peak irisin response in the serum of healthy adolescents compared to MCI exercise, whereas the induction of irisin was not evident in either exercise modality in obese or overweight adolescents. Differences in fat oxidation rates provide a possible explanation for the differences in irisin expression across exercise forms.

Not all results for the modulation of irisin in exercise are consistent. In 2015, the results of a meta-analysis covering 12 studies in 8 articles reported that chronic resistance training unexpectedly and significantly reduced circulating concentrations of irisin in a randomized controlled trial and that irisin also tended to be reduced in endurance exercise (Qiu et al., 2015). In a study conducted by Rodziewicz et al. (2020), the effects of a single incremental exercise on the plasma concentrations of irisin and BDNF were evaluated in subjects applying a treadmill for maximal fitness testing. While the study found a significant positive correlation between irisin concentrations and fasting glucose and insulin, no significant effect of irisin by exercise was observed (Rodziewicz et al., 2020). A recent systematic review and meta-analysis in 2021 compared the effect of exercise on irisin blood levels in 33 studies (Briken et al., 2016), with increased levels of irisin in 23 of these studies and decreased in another 10, with rather inconsistent results in individual studies. In the reported literature, exercises that have caused a decrease in irisin serum levels have included high-intensity circuit training (Yang et al., 2019), mixed physical training (Jandova et al., 2021), periodic sprint training (Gmiat et al., 2017), indoor aerobic training (Murawska-Cialowicz et al., 2015), and high-altitude mountaineering (Ozbay et al., 2020). Furthermore, even in the studies that have reported post-exercise upregulation of irisin, the timing of irisin upregulation is not same. More studies suggest that the upregulation of irisin occur immediately after exercise (Tsuchiya et al., 2015; Kubo et al., 2019; Cosio et al., 2021; Li H. et al., 2021; Tavassoli et al., 2022). In a clinical study involving gerontological neurodegenerative diseases, Briken et al. found that irisin was upregulated only immediately after exercise, while long-term exercise had no effect on serum irisin baseline (Sliwicka et al., 2017). However, it has also been reported that upregulation of irisin levels guided by short-term physical exercise in subcutaneous and visceral adipose tissue was able to persist for 1 week after cessation of the exercise and gradually returned to normal levels only 3 weeks after cessation of exercise (Tsuchiya et al., 2016). The cold environment can induce muscle tremors to achieve similar effects to exercise (Lee et al., 2014). A study in 2021 looked at changes in serum concentrations of irisin after ice swimming (Mu et al., 2020). Interestingly, when the cold environment was combined with exercise, unexpectedly, there was a significant decrease in serum irisin levels in winter swimmers (Mu et al., 2020), perhaps that may be due to the different tissue sources and metabolic environment of irisin. Furthermore, exercise does not always play a positive role in the body's metabolic processes and exercise may cause elevated expression of some inflammatory factors. It has been shown that excessive exercise triggers the activation of JNK/p38/ERK and ultimately significantly increases the levels of inflammatory factors, such as IL-1β, TNFa, and iNOS (Sun et al., 2017). Elevated inflammatory markers in serum are thought to be associated with aging, obesity, and chronic disease. Chronic peripheral inflammation may cause neurodegenerative lesions (Vints et al., 2022). Irisin is often thought to be an exercise-protective myokine that acts as an inflammatory resistance factor against inflammatory factors, used to reveal a possible link between exercise and the prevention of chronic disease (Martinez et al., 2018). A decrease in its expression is often used as a marker of chronic disease. However, as mentioned above, the release of irisin after exercise is closely related to the form and intensity of the exercise, as well as the physical condition and metabolic level of the subjects. The factors and mechanisms underlying exercise-induced irisin expression, the role of irisin in metabolic processes, and the relationship between irisin and other myokines or inflammatory factors need to be further investigated. Despite the partial controversy, the positive role of irisin in exercise-induced metabolic regulation is generally recognized.

## Irisin and aging

Exercise-induced expression of irisin is associated with resistance to aging. In fact, in an earlier study, Bostrom et al. reported that exercise was able to induce a significant increase in irisin levels in the circulating blood of elderly subjects (Bostrom et al., 2012). Analysis of gene microarray data by Timmons et al. (2012) indicated that exercise upregulated FNDC5 mRNA expression and that its elevated effect was more pronounced in older than in younger controls. In 2014, Aydin et al. compared serum irisin levels in young vs. older rats (Aydin et al., 2014). Their results showed that serum irisin was higher in young rats than in older rats in the quiet state. After 10 min of floating exercise, serum irisin increased in both young and old rats; however, the increase in irisin was significantly higher in the serum of young rats. Whereas, in another study, it was reported that baseline levels of irisin were significantly lower in older adults, the percentage level of increased irisin after acute exercise was not related to age or health (Huh et al., 2014). Although the effects of exercise modulation of irisin varied slightly in subjects of different ages, these studies suggest the potential of irisin in the alleviation of aging-related diseases. A study in rats compared the effects of age and exercise on irisin levels in heart, liver, and plasma samples from rats. The results showed that aging reduced the levels of irisin in the above tissues. Regular exercise increased irisin expression in all tissues analyzed compared to sedentary inactivity (Belviranli and Okudan, 2018). Recent studies have also shown that irisin is associated with improved neurocognitive performance in older adults and plays a key role in the facilitative effects of exercise on learning and memory (Babaei and Azari, 2021). More importantly, in a 2021 study, Colaianni et al. examined a series of patients undergoing total hip or knee arthroplasty. Serum irisin levels were negatively correlated with age and positively correlated with BMD. Further studies demonstrated that irisin treatment reduced the expression of the senescence marker p21 in osteoblasts *in vitro*, demonstrating the anti-aging ability of irisin in bone tissue (Colaianni et al., 2021).

Above, we mentioned that irisin plays a role in a number of aging-related diseases. Similarly, aging is generally considered to be a very important contributor to the development of osteoarthritis. Aging can cause a reduction in the capacity of articular cartilage cells to repair, destabilize the extracellular matrix of cartilage, and stimulate its degradation. At the same time, irisin causes remodeling of bone in the subchondral bone, causing changes in the mechanical stress of the joint, while causing the progression of osteoarthritis from multiple angles (Rahmati et al., 2017; Coryell et al., 2021). Although the mechanisms of aging in influencing disease development are not fully understood, signaling pathways associated with autophagy, apoptosis, and oxidative stress have been shown to be important in this (Rahmati et al., 2017; Coryell et al., 2021).

**Figure 2:**
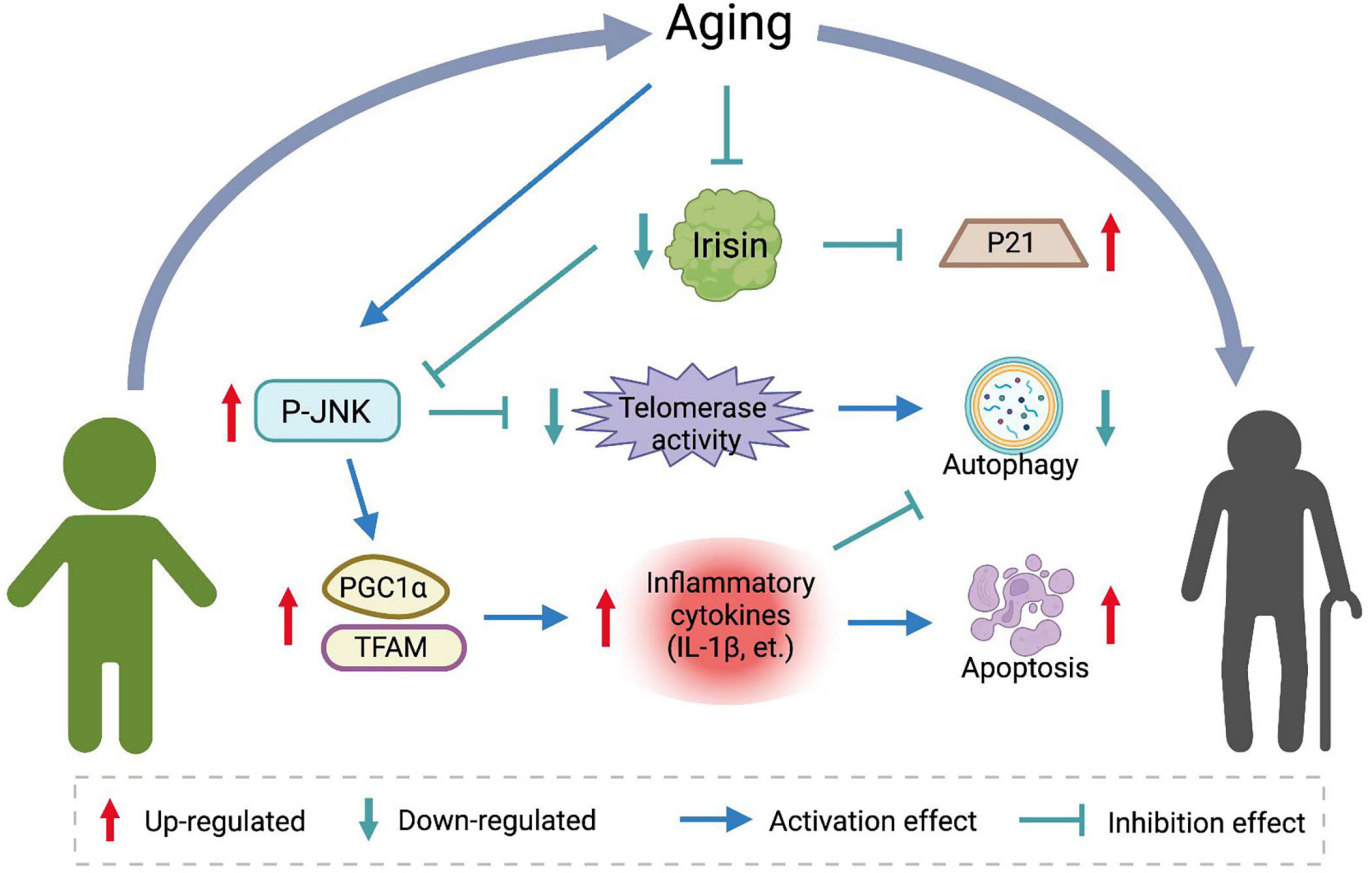
The role of irisin in aging resistance. Aging reduces baseline levels of irisin *in vivo*. Irisin inhibits JNK phosphorylation and affects telomerase activity and expression of inflammatory factors. Induces autophagy and apoptosis resistance and decreases markers of senescence.

In Belviranli and Okudan's study, irisin increased the action of the antioxidant enzyme superoxide dismutase. Exogenous irisin down-regulated the expression of apoptotic proteins in post-ischemic myocardium and inhibited inflammatory markers therein (Belviranli and Okudan, 2018). In another study, the PGC1a/FNDC5/irisin pathway supported exercise-induced selective autophagy, and irisin expression correlated with the expression of mitochondrial fission and mitochondrial phagocytosis markers in myotubes (He W. et al., 2020). A study by Bi et al. systematically analyzed the effects of irisin on the liver in an ischemia-reperfusion (IR) model. The results showed that age could influence the expression of irisin in liver tissue and the ability of liver tissue to repair the damage. At the same time, the levels of irisin expression, telomerase activity, autophagy, and mitochondrial function were lower in the liver tissues of aged rats. Exogenous irisin treatment in aged rats significantly reduced the mitochondrial function-related markers PGC1α and TFAM, and reduced inflammation, apoptosis, and oxidative stress in the injury model. Molecular experiments showed that irisin regulates telomerase activity through involvement in the MAPK pathway. Irisin inhibited the phosphorylation of JNK and increased telomerase activity in senescent hepatocytes to activate autophagy and improve mitochondrial function (Bi et al., 2020). In contrast, *in vitro* experiments, the JNK-MAPK inhibitor, an inhibitor of telomerase activity, eliminated the promotion of autophagy and the protective effect of irisin on mitochondrial function. A similar association was demonstrated in articular chondrocytes. In chondrocytes cultured in three dimensions *in vitro*, r-irisin treatment significantly reduced the phosphorylation levels of JNK, while significantly reducing inflammatory markers, such as IL-1β, and inhibiting the catabolism of chondrocytes with extracellular mechanisms (Vadala et al., 2020). Wang et al. (2020) also demonstrated that irisin promotes the protective effect on chondrocyte mitochondria by modulating the action of key factors, such as PGC1α, UCP1, and Sirt3, improving chondrocyte survival in an inflammatory environment and promoting ECM anabolism. The role of irisin in senescence resistance is shown in Figure 2.

## The effects of irisin on osteoarthritis

### Irisin and bone mineral density

Presumably, Irisin influences the progression of osteoarthritis by affecting bone mineral density (BMD). The increase in bone density requires the osteogenic effect of osteoblasts over the resorptive effect of osteoclastic bone. This process primarily involves both osteoblasts and osteoclasts, also known as osteogenic and osteoclastic cells. Osteoblasts are derived from mesenchymal stem cells (MSCs) in the bone marrow and eventually differentiate into osteoblasts, bone lining cells, or undergo apoptosis (Ponzetti and Rucci, 2021). Some studies have shown that progressive loss of bone, associated with the progression of osteoarthritis, is more pronounced especially in the elderly (Zhang et al., 2014; Fang et al., 2021). Xu et al. (2019) observed degenerative changes in articular cartilage. It was observed that the formation of bone redundancy was accompanied by a substantial bone loss in subchondral bone, with loss of normal bone structure in a rat OA model. Other partial findings also support the reduction of bone density in osteoarthritis (Schreiber et al., 2018; Muhlenfeld et al., 2021; Zhu Z. et al., 2021). A review in regard to the relationship between BMD and osteoarthritis of the knee by Choi et al. (2021) showed that patients with moderate and severe OA had significantly lower BMD than the normal ones. Patients with mild OA show an increase in BMD but this is most likely caused by a temporary increase in score due to high turnover of the osteoarthritic subchondral bone (Dequeker et al., 2003). Primary osteoarthritis includes loss of joint space, bone fragments, and cartilage sclerosis, which may lead to erroneous increases in BMD and become a confounding factor in the study.

At all ages, a significant correlation was shown between muscle mass, muscle strength, and bone density (Sutter et al., 2019; Chen F. et al., 2020; Lopes et al., 2021). Irisin, a myokine induced during exercise, is thought to be an osteoprotective factor that acts on bone and affects bone density. The expression of irisin is positively correlated with bone health (Colaianni et al., 2019), and the related studies have been widely reported. In earlier studies, circulating irisin showed a negative association with the incidence of osteoporotic fractures in post-menopausal women with low bone mass (Anastasilakis et al., 2014; Yan et al., 2018). In a study of serum irisin levels in white football players, Colaianni et al. used x-ray bone densitometry to measure the BMD in the whole body and in different bone subregions (head, arms, legs, etc.) and compared the relationship between irisin concentrations and whole-body BMD. The results showed that irisin was linearly correlated with BMD at different bone sites and that there was a systematic association between circulating irisin and bone mass (Colaianni et al., 2017a). Zhang et al. (2020) also demonstrated a positive correlation between irisin levels and BMD in older Chinese men in 2020, and these results supported the idea that irisin exerts a protective effect on bone tissue. In 2022, Wang et al. investigated the relationship between serum levels of irisin and BMD in patients with new-onset type 2 diabetes and observed that decreased serum levels of irisin were negatively associated with BMD. The hindlimb suspension mouse model, a widely accepted mouse model that simulates weightlessness, was used to determine the effects of unloading conditions on the performance of the musculoskeletal system (Morey-Holton et al., 2005). Bone loss induced by lack of mechanical loading was effectively prevented by the recombinant iris, and the density of femur and tibia in mice treated with recombinant iris for 4 weeks was even higher than in vehicle-treated mice (Colaianni et al., 2017b). Irisin similarly prevents bone loss in a devitalized mouse model (Luo et al., 2020). After Luo et al. treated de-ovulated mice with r-Irisin for 5 weeks, mice in the irisin-treated group exhibited a significant improvement in bone microarchitecture, bone density, and bone volume to tissue volume ratio relative to the control group. Histomorphometric analysis also showed that r-irisin increased the number of osteoblasts and decreased the number of osteoclasts. This result is consistent with the results in the deovulated rat model (Morgan et al., 2021). Irisin protected normal bone structure, maintained bone density, and maintained bone metabolic homeostasis. To further validate the relationship between irisin and bone metabolism, Zhu's team constructed FNDC5/irisin knockout mice in 2021 (Zhu X. et al., 2021) and observed a significant decrease in BMD and delayed bone development and mineralization in knockout mice during development and into adulthood. Knockout of irisin impaired the increase in bone thickness in mice induced by wheel running exercise and reduced the level of fat browning *in vivo*. Notably, the de-ovulatory model is a common means used to induce osteoporosis (Kawao et al., 2021), with osteoarthritis (Xu et al., 2019). In humans, more than 30% of menopausal women are affected by reduced bone mass and deterioration of bone tissue microarchitecture (Rossi et al., 2018), and levels of related hormones such as estrogen are associated with bone density. As mentioned above, exercise-induced expression of irisin ameliorates osteoporosis and osteoarthritis induced by the de-ovulatory model (Morris et al., 2018; Kawao et al., 2021). Another study showed that *in vitro*, irisin treatment significantly upregulated estrogen receptor alpha in cells (Yang et al., 2021). However, although the two together affect metabolic pathways, including insulin resistance (Park et al., 2013; Li et al., 2019), there are no clear reports on whether there is a direct link between the regulatory effects of irisin and the estrogen pathway.

We have described the existence of a relationship between OA and bone density above (Dequeker et al., 2003; Choi et al., 2021), and the role of irisin in modulating bone density to improve bone quality helps to explain the effects of mechanical signal stimulation on inhibiting the progression of osteoarthritis. In 2020, a study by He et al. showed that irisin rescued bone volume fraction and trabecular number in ACLT model mice and improved bone density by reducing osteoblast apoptosis. So that cartilage structure is stabilized that may indirectly stimulate the induction of chondrocyte proliferation and improve the progression of osteoarthritis through mechanical signaling of bone *in vivo* (He Z. et al., 2020). Interestingly, Wang et al. observed a decrease in serum levels of irisin in type 2 diabetic patients in parallel with a study that observed a downregulation of bone transformation markers, including osteocalcin (Wang et al., 2022). Osteocalcin—a protein specifically secreted by osteoblasts—is an important bone factor, the expression of which has been reported to be associated with osteoarthritis (Kalichman and Kobyliansky, 2010; Tarquini et al., 2017). Both irisin and osteocalcin are exercise-induced cytokines and are important components of the constitutive muscle-bone crosstalk (Kirk et al., 2020). Wang's results provide us with the possibility that irisin indirectly regulates osteoarthritis by causing alterations in the expression of bone-derived biochemical signals, and whether there is a direct regulatory effect between irisin and osteocalcin remains to be further investigated.

### Irisin and bone metabolism

The metabolic regulation of irisin in bone is achieved in part by binding to integrin proteins to activate the intracellular wnt/β-catenin and ERK/MAPK signaling pathways. Changes in bone metabolism are key to affecting bone density. Changes in the balance between bone resorption and bone formation lead to changes in bone mass and affect bone remodeling (Kobayashi et al., 2016; Shen et al., 2020), thus affecting the mechanical stress state and structural stability of joints. Bone loss and osteocyte death caused by imbalances in bone metabolism lead to bone diseases, such as osteoporosis (Kim et al., 2013), femoral head necrosis (Chen et al., 2019), and the rapid progression to osteoarthritis.

Studies have shown that integrin proteins are the primary receptors for irisin in osteogenic lineage cells. Integrins are heterodimeric transmembrane proteins composed of alpha and beta subunits that mediate cell adhesion, influence cell development, immunity, and participate in hemostatic responses and wound healing. Integrins also act as signal transduction receptors, triggering a series of intracellular signaling pathways that are involved in controlling cell survival, proliferation, and regulating tissue metabolic homeostasis, as well as the progression of a variety of diseases (Barczyk et al., 2010; Ginsberg, 2014). In 2018, Kim et al. found that, as a muscle factor with increased expression induced by exercise, irisin caused the phosphorylation of FAK and Zyxin, the main downstream proteins in the integrin protein signaling pathway in human osteoid cells. Quantitative proteomic analysis confirmed the elevated expression of several integrin family members in response to irisin induction. The αV integrin protein is the possible receptor of irisin. Both αV/β5 and αV/β1 show a strong affinity with irisin. Mass spectrometry analysis further confirmed the direct binding between irisin and integrin αV/β5, while identifying the structural sequences (amino acids 60–76 and 101–118) involved in the binding (Kim et al., 2018). Kim et al. further blocked irisin-induced signals using inhibitors of various αV integrin complexes. These results confirm the important role of the integrin αV complex as an irisin receptor in osteoblasts. It also suggests that the integrin αV complex may also function as an irisin receptor in adipose tissue, mediating irisin-induced lipid metabolic processes. The role between irisin and integrins contributes to the understanding of the regulation of bone metabolism by irisin and the interpretation of the role of exercise in human health.

**Figure 3:**
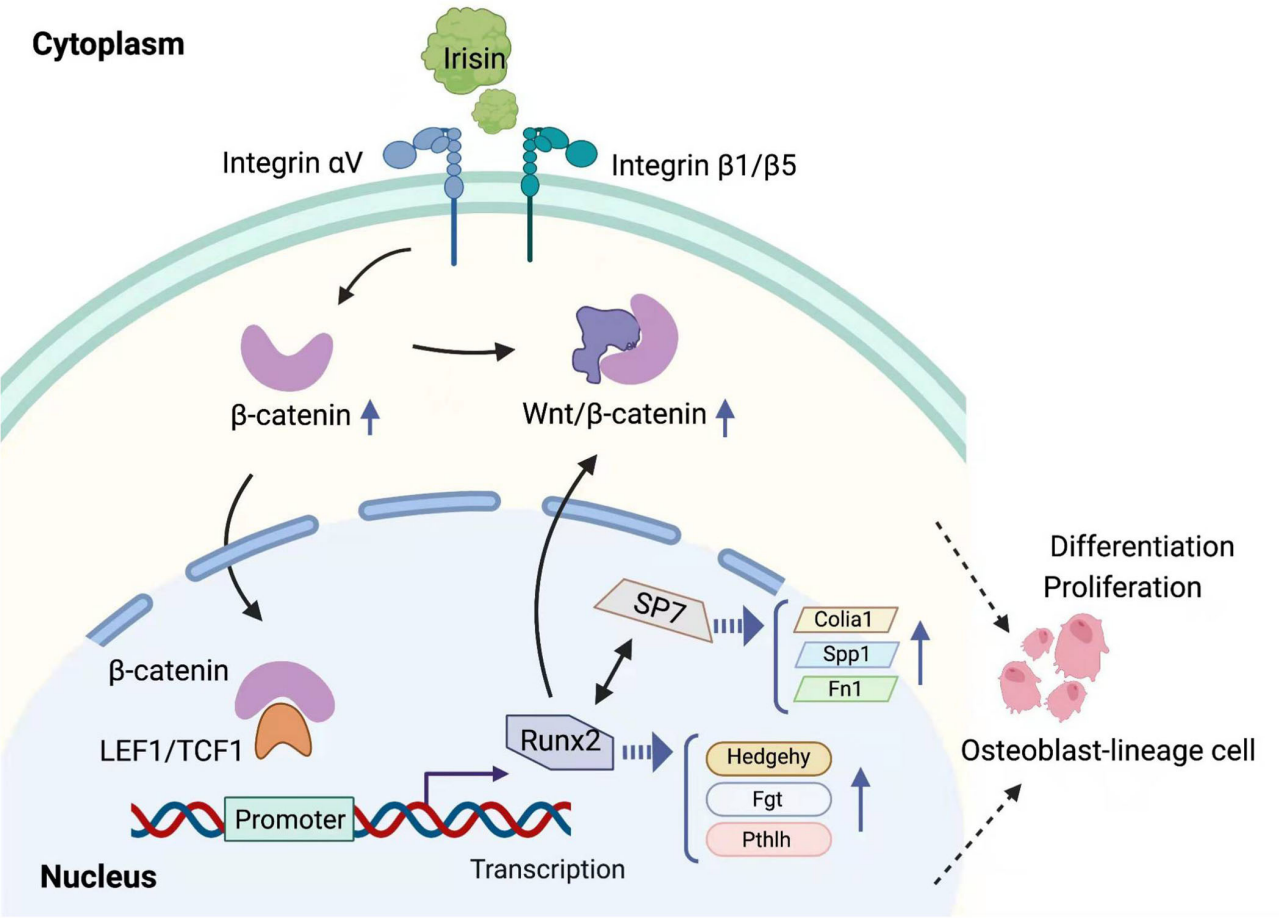
Mechanisms of regulation of the Wnt/β-catenin pathway by irisin in osteogenic lineage cells. Irisin affects various steps of the Wnt/β-catenin pathway by binding to integrins and causing the accumulation of intracellular β-catenin.

Irisin is induced by exercise, and the mechanism of its intracellular regulation of the WNT pathway in osteoblast-lineage cells is shown in Figure 3. Physical exercise can resist aging and mineral loss in bones caused by aging *via* regulating the balance between bone resorption and bone formation, which is associated with higher BMD. Current studies have demonstrated that the differentiation process of osteoblasts is regulated by the WNT signaling pathway and that RUNX2 is an important transcription factor in the osteogenesis process (Ponzetti and Rucci, 2021). Wnt/β-catenin pathway plays a key role in this metabolic balance regulated by exercise (Faienza et al., 2020). Previous studies have demonstrated that members of the integrin family (including αV/β1) can activate WNT1 expression and thus form positive feedback in the Wnt/β-catenin pathway by causing the accumulation of β-catenin (Du J. et al., 2016). In 2021, Liu et al. reviewed some of the studies in which exercise promoted the expression of irisin and found that the anti-inflammatory effects induced by irisin in bone tissue were associated with the activation of the classical Wnt signaling pathway (Liu et al., 2021). In a study by Zhu X. et al. (2021), FNDC5/irisin-deficient mice consistently showed reduced BMD. Irisin deficiency inhibited osteoblastogenesis and increased osteoclastogenesis, and a concomitant decrease in adipose tissue browning was observed in model mice. Elevated concentrations of irisin were induced by voluntary rotational exercise and upregulated total protein levels of β-catenin in bone marrow mesenchymal stem cells. The intranuclear expression of irisin activated Wnt/β-catenin pathway and recombinant irisin (r-irisin) induced osteoblast differentiation. It also inhibited the differentiation ability of osteoblasts and contributed to healthy bone anabolism. The result is consistent with the study by Luo et al. (2020). The accumulation of β-catenin promotes the expression of an important transcription factor, Runx2. β-catenin can bind to the promoter sequences of Runx2 and activate its transcription. Overexpression of Runx2 upregulates the expression of Tcf7, Wnt10b, and Wnt1 genes, and realized their mutual regulation with the wnt/β-catenin signaling pathway. Runx2 enhanced the proliferation of pluripotent mesenchymal cells and induced their transformation into pre-osteoblasts through the regulation of the genes involved in hedgehog, wnt, Fgf, and Pthlh signaling pathways (Morris et al., 2018; Qin et al., 2019; Chen D. et al., 2020). The expression of genes, such as Col1a1, Spp1, and Fn1, was upregulated (Komori, 2018, 2019; Chen D. et al., 2020). Taken together, the irisin-integrin-wnt/β-catenin-Runx2 interplay may constitute a signaling axis, providing a possible explanation for the mechanism of action of irisin-induced osteoblast differentiation and improved BMD.

**Figure 4:**
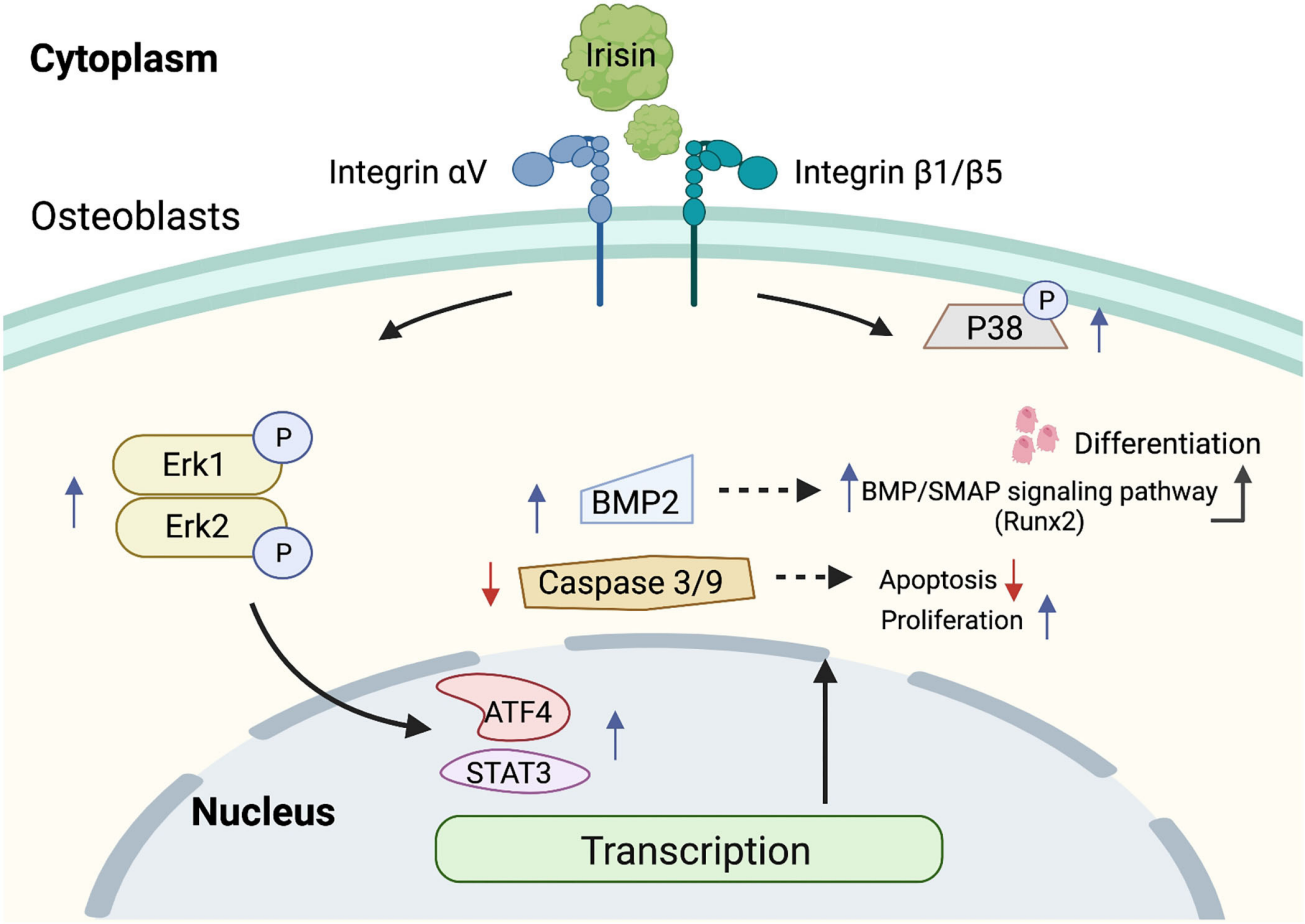
Mechanism of regulation of ERK MAPK signaling pathway by irisin. Irisin binds to integrins, increases the amount of p-P38 and p-Erk1/2, activates the ERK MAPK signaling pathway, resists apoptosis, promotes proliferation, and induces osteoblast differentiation.

Figure 4 demonstrates the activation of the ERK/MAPK signaling pathway mediated by irisin-binding integrins. The integrin family can activate the expression of the ERK/MAPK signaling pathway (Sheng et al., 2017; Morris et al., 2018). Colaianni et al. found that r-Irisin was able to rapidly induce phosphorylation of Erk and upregulate the expression levels of osteoblast marker genes, such as Atf4 and Runx2 (Colaianni et al., 2015). r-Irisin activated MAP kinases Erk1 and Erk2 and increased the expression of transcription factor Atf4 and down-regulated apoptotic factors caspase-9 and caspase-3 in osteoporotic mouse osteoblasts, MLO-Y4, *in vitro*, which in turn exerted anti-apoptotic effects (Storlino et al., 2020). Recombinant irisin with a conditioned medium containing irisin significantly increased the number of phosphorylated P38 (p-P38) and phosphorylated ERK (p-ERK) in rat primary osteoblasts, but had no effect on total protein. The inhibition of phosphorylated P38 with phosphorylated ERK, along with the inhibition of irisin-induced Runx2 expression, inhibited osteoblast proliferation (Qiao et al., 2016). Recently, Xue et al. showed that irisin promoted phosphorylation of Erk1/2 *via* integrin receptor αV, which in turn increased phosphorylation of STAT3 and promoted increased expression of BMP2. The binding of BMP2 to the membrane surface receptor BMPR2 activated the BMP/SMAD signaling pathway, ultimately promoting osteogenic differentiation (Xue et al., 2022). The role of irisin in the P38/ERK MAPK signaling pathway remains somewhat unresolved, and the regulation of ERK and P38 phosphorylation by irisin may be different in other tissues than in osteoblasts. For example, the investigators demonstrated in macrophages that irisin caused a decrease in the release of pro-inflammatory cytokines, such as IL-1β, TNFα, and IL-6. This was associated with the activation of phosphorylation of the MAPK signaling pathway. Here, irisin significantly reduced the phosphorylation levels of JNK and ERK, but had no effect on p-p38 (Mazur-Bialy et al., 2017). Although its mechanism of action in osteoblasts may differ, irisin exhibits a distinct anti-inflammatory profile. In another study, irisin promoted the proliferation of C2C12 cells *in vitro* by activating the ERK signaling pathway. This is a myogenic cell and treatment with irisin increased ERK; phosphorylation levels in a dose-dependent manner, while having no significant effect on p38 phosphorylation in the short term (Lee et al., 2019). In conclusion, the irisin/P38/ERK MAPK signaling pathway plays a role in resisting osteoblast apoptosis and promoting osteoblast proliferation, enhancing bone formation, and improving bone density and quality.

In addition, the reciprocal regulatory role of irisin in the AMPK signaling pathway has been reported in many tissues. Exercise-induced elevation of irisin is associated with AMPK activity in the kidney. Irisin activates AMPK in renal tubular cells, inhibits the expression of inflammatory factors in the kidney, and exerts a protective effect on the kidney (Formigari et al., 2022). Studies have shown that the irisin precursor protein FNDC5 attenuates inflammation and insulin resistance in adipose tissue through the AMPK-mediated polarization of obese macrophages (Xiong et al., 2018). In bone, recent studies have shown that the induction of osteogenesis by irisin is also associated with macrophage polarization (Ye et al., 2020). Irisin treatment promoted the polarization of M0 macrophages to the M2 phenotype and facilitated osteogenesis. siRNA for the AMPK subunit AMPK-α significantly abolished the Irisin-induced M2 phenotype switch and reduced the osteogenic capacity of Irisin-treated macrophages. This result suggests that the induction of polarization of macrophages by irisin may be achieved through activation of the AMPK signaling pathway. At present, there are relatively few studies of irisin and the AMPK signaling pathway in bone. The regulatory mechanisms associated with irisin in bone metabolism need to be further investigated.

### Irisin and cartilage

Along with regulating bone density, irisin can directly influence the development of osteoarthritis by regulating the proliferation and apoptosis of chondrocytes and maintaining the homeostasis of the extrachondral matrix. Chondrocytes are derived from mesenchymal cells in the early embryo (Lefebvre and Smits, 2005). At the onset of skeletogenesis, mesenchymal precursor cells undergo chondrogenesis and differentiate into prechondrocytes. Of these, early chondrogenic cells located within the joint cavity develop into articular chondrogenic cells and eventually differentiate into articular chondrocytes. Articular cartilage is a special transparent tissue that covers the surface of joint bones and is composed of chondrocytes and an extracellular matrix (Madry et al., 2010). Chondrocytes are surrounded by a rich layer of extracellular matrix ECM that regulates chondrocyte differentiation and activity while maintaining the biomechanical properties of the tissue (Lefebvre and Smits, 2005). As a common joint disease, damage to articular cartilage is a key feature of osteoarthritis, including inflammation-induced apoptosis of chondrocytes and degradation of the extracellular matrix (Jang et al., 2021). The association between irisin concentrations and osteoarthritis was first analyzed in a study of blood and synovial fluid (SF) from patients with osteoarthritis of the knee. The results showed that irisin concentrations in serum and SF decreased with increasing Kellgren-Lawrence classification and that irisin concentrations were negatively correlated with the imaging severity of osteoarthritis. The expression of C-reactive protein (CRP) in serum was increased with the decrease of irisin concentration (Mao et al., 2016).

Exercise alleviates the inflammatory state of osteoarthritis, and irisin expression shows a correlation with resistance to inflammation. In a recent study, moderate-intensity exercise significantly increased serum protein levels of irisin and the anti-inflammatory factor IL-10, which inhibited expression of the inflammatory factor TNF-α and improved the osteoarthritis index in older women with sarcopenia and OA (Park et al., 2021). In the last 2 years, further studies on the protective effects of irisin on cartilage tissue in osteoarthritis have been reported. *In vitro*, r-irisin increased type II collagen levels in three-dimensionally cultured human osteoarthritic chondrocytes, while decreasing the expression of the cartilage ossification marker type X collagen. The signals improved cartilage homeostasis. In contrast to its report in osteoblasts, r-irisin treatment reduced the phosphorylation levels of p38, Akt, JNK, and NF-κB but not phosphorylated ERK in chondrocytes over a short period of time (Vadala et al., 2020). These findings signify the possible cellular specificity of irisin signaling in osteoblasts and chondrocytes. Irisin inhibited the differentiation of chondrocytes to osteoblasts, reduced the expression of inflammatory factors IL-1 and IL-6, inhibited chondrocyte apoptosis, downregulated the metalloproteinases MMP-1 and MMP-13, maintained the protective role of the extracellular matrix in cartilage, and promoted chondrocyte proliferation (Vadala et al., 2020). Wang et al. also demonstrated that irisin injection improved gait and inhibited IL-1β-mediated loss of the autophagic markers Atg4 and Atg12, as well as p62 in DMM mice. Irisin inhibits chondrocyte apoptosis and promotes extracellular matrix accumulation by improving membrane potential and mitochondrial biogenesis in chondrocytes to protect articular cartilage and slow the development of OA (Wang et al., 2020).

However, there are conflicting reports on the ability of irisin to promote chondrocyte proliferation. He et al. observed a significant increase in the proportion of hyaline cartilage with reduced calcification of tibial cartilage at the knee joint in an ACLT model mouse. The results showed that there were no changes in the ability of chondrocytes to proliferate after being administered intravenously with irisin that was observed in an *in vitro* pulling assay that simulated exercise. Accordingly, He et al. concluded that the alleviating effect of irisin on osteoarthritis was mainly achieved by reducing the apoptosis of bone cells in subchondral bone and improving the microstructure of subchondral bone (He Z. et al., 2020). To further clarify the role of irisin in cartilage tissue, in 2021, Li et al. cultured and characterized irisin KI with KO in transgenic mice. *In vivo*, irisin knockout mice exhibited more severe osteoarthritic features following DMM modeling, whereas KI mice with intra-articular injection of irisin significantly resisted DMM-induced progression of osteoarthritis. In experiments with primary chondrocytes cultured *in vitro*, cell proliferation in irisin KI mice was significantly increased in both normal and IL-1β-induced inflammatory states, while KO mouse-derived chondrocytes had decreased proliferative capacity, and the addition of r-irisin was able to reverse the proliferative capacity of KO cells. Irisin reduced the gene expression levels of inflammatory factors and inflammatory mediators in chondrocytes and upregulated COL2a1, aggrecan, and SOX9 expression. These experiment results clarify the role of irisin in promoting chondrocyte proliferation Li X. et al. (2021). We speculate that differences in the intensity settings of mechanical pulling *in vitro* and the forms of cell culture in the different studies may have contributed to the differences in the results of the different studies. Primary chondrocytes cultured in 3D can better simulate the effect of irisin *in vivo*. In a recent study, Jia et al. compared the effects of low, moderate, or high-intensity treadmill exercise with the effects on the concentration levels of irisin in the synovial fluid of SD rats (Jia et al., 2022). This study demonstrated that exercise of appropriate intensity maintained high circulating levels of irisin, significantly increased the concentration of irisin in synovial fluid, alleviated inflammation and scorching of chondrocytes, reduced OA cartilage damage in a rat model, and achieved therapeutic effects in osteoarthritis. In contrast, high-intensity exercise may cause excessive mechanical stimulation, resulting in damage that outweighs the therapeutic effect of increased irisin concentrations and therefore exacerbates the progression of OA.

These results suggest that irisin, as an exercise-induced muscle factor, may act directly on cartilage tissue and influence the disease process of knee OA by inhibiting inflammation. The ameliorative effect of irisin on osteoarthritis is mediated by its modulation of subchondral bone mass in conjunction with its direct intervention on cartilage tissue metabolism.

### Irisin and cartilage metabolism

**Figure 5:**
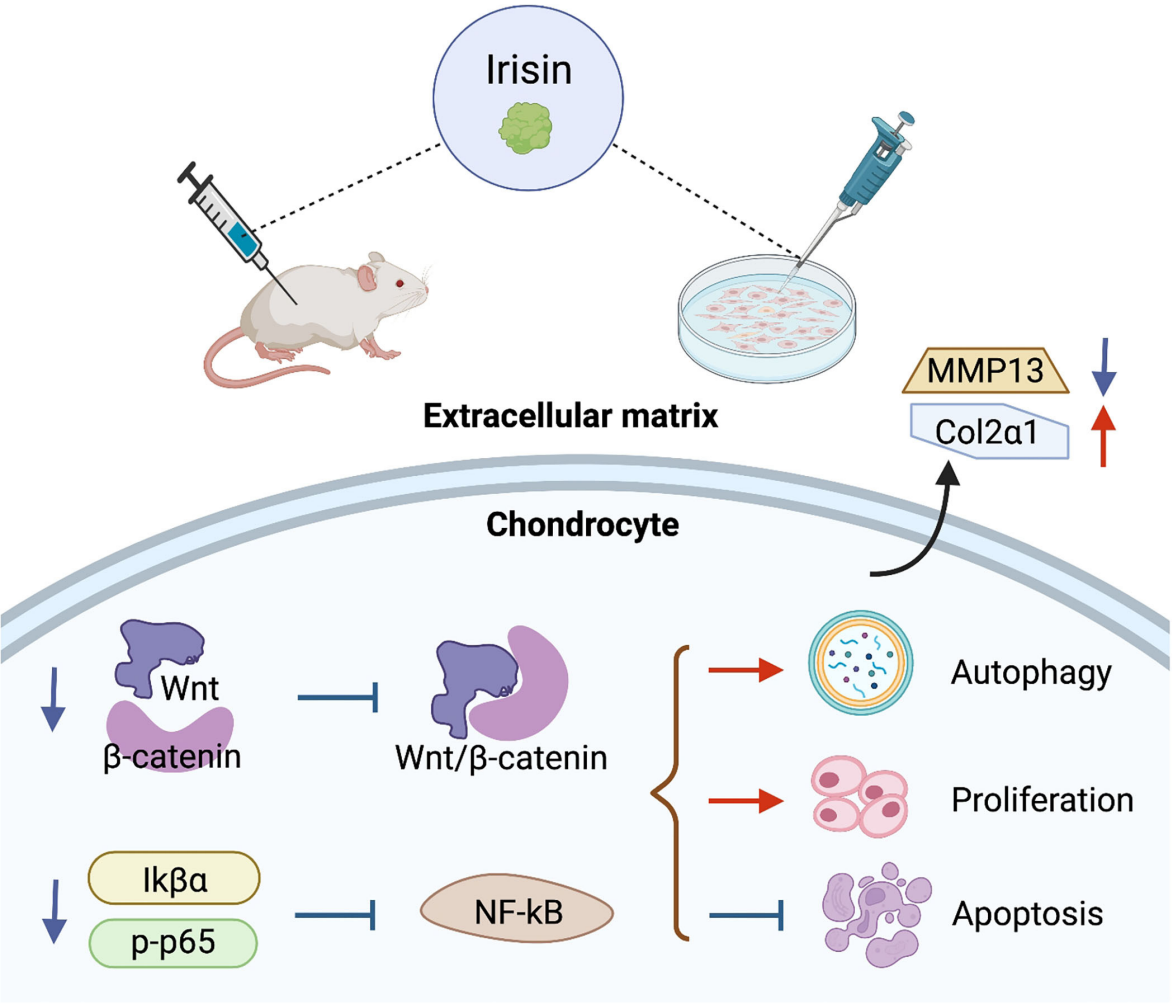
Regulatory role of irisin in chondrocyte metabolism. Irisin inhibits the activation of Wnt/β-catenin and NF-κB signaling pathways in osteoarthritic chondrocytes, promotes cell autophagy and proliferation, inhibits apoptosis, and improves the stability of the extracellular matrix.

High expression of irisin inhibits the activation of chondrocyte Wnt/β-catenin and NF-κB signaling pathways in osteoarthritis. The regulation within chondrocytes is shown in Figure 5. The metabolic imbalance between anabolic and catabolic factors produced by chondrocytes leads to the degradation and destruction of cartilage. This is an important cause of chondrocyte apoptosis, as well as degradation of extrachondral mechanisms, ultimately leading to osteoarthritis (Messina et al., 2019; Oliviero and Ramonda, 2021).

Activation of Wnt/β-catenin and NF-κB signaling pathways in chondrocytes regulates chondrocyte metabolism and plays an important role in OA progression (Zhou et al., 2017; Choi et al., 2019). Among these, the Wnt/β-catenin signaling pathway regulates the development of arthritis and mediates the high expression of downstream effectors, such as mmp family members, adamts, acan, and Col2a1 genes and inflammation-related factors in chondrocytes with osteoarthritis (Zhou et al., 2017). The strong activation of typical Wnt signaling exhibited joint damage due to DMM surgery in chondrocytes. In transgenic mice with stable mutations in β-catenin, the model showed a significant progressive loss of articular cartilage as β-catenin expression in cartilage was upregulated. Inhibition of the activation state of the Wnt pathway in cartilage tissue alleviates OA symptoms and mitigates cartilage loss in a surgically induced mouse model of OA (Lietman et al., 2018). NF-κB is aberrantly activated in OA and the associated signals are involved in a variety of biological processes, including chondrocyte apoptosis, inflammatory factor release, and extracellular matrix degradation (Chen Z. et al., 2020) Tumor necrosis factor-alpha (TNF-α) is an important cytokine (Cao et al., 2021), and studies have shown that IL-1β-induced TNF-α expression is significantly upregulated in chondrocytes and activates the NF-kB pathway by increasing p-IkBa and p-p65 (Li et al., 2019) NF-kB pathway (Li Z. et al., 2021).

Irisin is a myokine that can be upregulated after being induced during exercise. Wang et al.'s (2020) study confirmed that the irisin signaling pathway plays a protective role on the mitochondria of chondrocytes, inhibiting apoptosis and preventing oxidative damage in inflammatory chondrocytes, along with key regulators, including Sirt3, a signaling factor closely associated with the activation of Wnt pathway. Li et al. induced osteosarcoma cells with IL-1β to simulate OA *in vitro*. The results showed that irisin treatment significantly inhibited the protein and mRNA levels of WnT-1 and β-catenin. Irisin intervention also significantly reduced the induction effect of LiCl on β-catenin. Irisin inhibited Wnt/β-catenin pathway in chondrocytes. The expression of MMP-13 and other metal matrix proteases in cartilage tissues was down-regulated, and the protein level of type II collagen in IL-1β-induced osteosarcoma cells was reversed. Irisin showed strong resistance to inflammation (Li et al., 2020). On the other hand, irisin reduced TNF-α expression in chondrocytes cultured *in vitro* (Li X. et al., 2021) demonstrated that irisin reversed IL-1β-induced IkBa expression and p65 phosphorylation levels in osteosarcoma cells. Irisin inhibited cytoplasmic p-p65 and down-regulated NF-κB pathway activity in chondrocytes (Li et al., 2020). *In vitro* morphological observations demonstrated that upregulation of irisin concentrations ameliorated chondrocyte scorch death, an important pro-inflammatory programmed cell death (Jia et al., 2022). In Jia et al.'s study, irisin pretreatment blocked IL-1β-induced NF-κB/p65 nuclear translocation and enhanced chondrocyte-specific collagen II expression, while inhibiting nod-like receptor protein-3 (NLRP3)/caspase-1 activity. The results of both biochemical analysis and biochemical indexes indicated that irisin ameliorated osteoarthritis by inhibiting the activation of PI3K/Akt/NF-κB cascade reaction and suppressing inflammation-induced chondrocyte scorching (Jia et al., 2022).

Irisin exhibited a protective effect on cartilage by inhibiting the activation of Wnt/β-catenin and NF-κB signaling pathways, reducing matrix metalloproteinase expression, enhancing extracellular matrix stability, downregulating inflammatory factors in chondrocytes, inhibiting apoptosis, and promoting chondrocyte proliferation. Notably, the regulation of key factors of the Wnt/β-catenin pathway by irisin in chondrocytes showed opposite characteristics to its promotion of the Wnt/β-catenin pathway in osteoblastic lineage cells that reflect the cellular specificity of irisin.

## Prospect

It is expected that in the future, research on irisin, a muscle factor induced by exercise, will help us to further understand and explain the beneficial effects of exercise in the maintenance of physical health, particularly in the fight against aging and age-related degenerative diseases. As shown in Supplementary Table 1, the effect of different types of exercise on the induction of irisin expression is clearly inconsistent, with resistance exercise being more significant in increasing irisin levels, and a great deal of research has been done to explain the mechanisms underlying this phenomenon. The FITT principle, which includes frequency, intensity, time, and type, is a standard prescription for physical activity programs as newly defined by the American College of Sports Medicine (ACSM) (DeSimone, 2019). Exercise protocols based on the FITT principles hold the promise of resolving the contradictory results that have emerged from past studies of exercise modulation of irisin and will help to further clarify the modulatory effects of exercise on irisin in the future. The roles of irisin in bone and cartilage tissue and the mechanisms that trigger it (as shown in Figures 3–5) have received a great deal of attention in the exercise treatment of osteoarthritis. In the past 2 years, studies involving the roles of irisin in osteoarthritis have increased rapidly, revealing the regulation of bone density (Zhang et al., 2020; Wang et al., 2022), resistance to inflammatory factors (Vadala et al., 2020; Wang et al., 2020), and the protective effects of irisin on articular cartilage (Li X. et al., 2021; Jia et al., 2022). However, the research on related pathways is still not thorough, and many important nodes are missing. For example, what is the receptor of irisin in chondrocytes? Irisin plays different roles in bone and cartilage tissues, and the reasons for its obvious tissue specificity and its role in coordinating the metabolic balance between bone tissue, cartilage tissue, and other body tissues are all problems that need to be solved in the future.

## Conclusion

Irisin, a myokine secreted by skeletal muscle in response to exercise stimulation, is involved in resistance to aging and its discovery explains the protective mechanism of exercise in bone and cartilage tissues. Irisin has been shown to promote osteoblast differentiation and proliferation in bone, increase bone density, improve bone quality, and enhance the mechanical support of cartilage by subchondral bone; on the other hand, irisin promotes chondrocyte proliferation, reduces the secretion of inflammatory factors and matrix metalloproteinases in chondrocytes, inhibits chondrocyte apoptosis, and strengthens the stability of the extrachondral matrix, which has great potential in the prevention and treatment of osteoarthritis. In conclusion, this study shows that irisin is a very useful tool in the prevention and treatment of osteoarthritis. In conclusion, this study suggests that irisin may be a new marker signal and therapeutic target in osteoarthritis. Further research into the effects of exercise form and intensity on the induction of irisin expression, as well as the mechanisms regulating the effects of irisin in bone density and cartilage metabolism, will help to implement better prevention and treatment modalities for osteoarthritis.

## Author contributions

X-aZ, ZW, and KN designed the research and collected the materials. X-aZ provided conceptual and funding support for this article. X-aZ and ZW reviewed and revised the manuscript. KN wrote and amended the manuscript. All authors contributed to the article and approved the submitted version.

## Funding

This work was supported by the Innovative Talents Support Program for Universities of Liaoning Province, No. WR2019024.

## Conflict of interest

The authors declare that the research was conducted in the absence of any commercial or financial relationships that could be construed as a potential conflict of interest.

## Publisher's note

All claims expressed in this article are solely those of the authors and do not necessarily represent those of their affiliated organizations, or those of the publisher, the editors and the reviewers. Any product that may be evaluated in this article, or claim that may be made by its manufacturer, is not guaranteed or endorsed by the publisher.
