# Supplementary material for: Exercise-induced modulation of myokine irisin in bone and cartilage tissue—Positive effects on osteoarthritis: A narrative review
Source: Front Aging Neurosci. 2022 Aug 19;14:934406. doi: 10.3389/fnagi.2022.934406 (PMC9439853; doi:10.3389/fnagi.2022.934406)
Supplement: Supplementary file 1 [file Table_1.DOCX]

Supplementary Material

**Supplementary Table 1** ｜Induction of irisin by different exercise

| **Study** | **Object of study** | **Exercise** | | | | | **Timing of blood collection** | **Irisin after exercise** |
| --- | --- | --- | --- | --- | --- | --- | --- | --- |
|  |  | **Type** | **Forms** | **Frequency** | **Intensity** | **Time** |  |  |
| (Wrann et al.,2013) | Female C57B1/6 mice | Endurance exercise | Running wheels | Free | Free | 30 days | 10 h after last bout of exercise | Increase* |
| (Tavassoli et al.,2022) | Male Wistar rats | Resistance exercise | Climb a vertical ladder | 3 day/week | 85%incline,50% -100% of the animal’s total weight | 12 weeks | After 10–12 h of night fasting | Decrease* |
|  |  |  |  |  |  |  |  |  |
| (Kubo et al.,2019) | C57BL/6 male mice | Aerobic exercise | Motor-driven treadmill | Once a day , five times per week | 18 m/min | 30 min per session,12 week | 3 h after the last exercise session | Elevated* |
| (Li et al.,2021) | Male C57BL/6J wildtype mices | Aerobic exercise | Treadmill training | 5 days per week | 12 m/min | 60 min per day;4 week | Unclear | Increase**, resistance exercise is the most significant |
|  |  | Resistance exercise | Climb a vertical ladder | 8 rounds per day,3 times per round,  1 min rest between each round,5 days per week | 85%incline, 0% to 75% of body weight increased by 10% a day | 4 weeks |  |  |
| (Tsuchiya et al.,2015) | Physically active males | Resistance exercise | Weight-stack machine and/or  incremental pedaling test | 3 times, separated by at least 1 week | 65% of 1RM and/or 65% of VO2max | The total time for 60 min | Before exercise and at  0, 0.5, 1, 2, 3, 4, and 6 h after exercise | Significantly elevated * at 1 h after resistance exercise |
|  |  | Endurance exercise |  |  |  |  |  |  |
|  |  | Resistance exercise and  endurance exercise |  |  |  |  |  |  |
| (Huh et al.,2014) | Healthy men | Aerobic exercise | Treadmill training | Once | 70%–75% of VO2max;90% of VO2max | 45 min,Exhaustion | 10 –15 min after exercise | Lower in physically active** |
|  | Healthy adolescent swimmers | CME | 2000m of continuous freestyle swimming | Once | 1.21±0.02 m/s | 27 min 37 seconds ±22 seconds | Pre-exercise, in less than 5 min;  postexercise,0h,1h and 24h | Increased immediately after exercise* |
|  |  | HIIE | 50m maximal freestyle swimming | Six times every five min | 1.56 ±0.02 m/s. | 32.23 ±0.47 seconds/each time |  | Significantly upregulated** |
| (Colpitts et al.,2022) | Youth between 12 and 18 years old | CME | Cycle ergometer | Once | 50% heart rate reserve | 35 min | Pre-exercise(0min), during the exercise at 7, 14, 21, 28, immediately post-exercise at 35min. | No difference between weight; higher after HIIE* |
|  |  | HIIE |  | Once | 50% HR for 5 min followed  by 2min of cycling at 85%–90% HR | 5 times within 35 min |  |  |
|  |  |  |  |  |  |  |  |  |
| (Rodziewicz et al.,2020) | Men aged 58 ± 3.6 years | Incremental exercise | Mechanical treadmill | Once | 8 km/h, then increased by 2 km every 3 min | Exhaustion | Before and 10 min  after incremental exercise | Non-significant; decreased (18.4%) in the untrained group and increased (12.5%)  in the trained group |
|  |  |  |  |  |  |  |  |  |
| (Briken et al.,2016) | Patients with multiple sclerosis | Acute exercise | Standardized bicycle ergometry performance test | One time before the Long term training and one time after the end | 25 W with an incremental increase of 12.5 W/min  or 8 W with an incremental increase of 8 W/min. | Exhaustion,average 10–20 min | Directly after and  30 min after termination | No significant effects |
|  |  | Endurance exercise | Arm ergometry, rowing  and bicycle ergometry | 2-3 times a week | Tailored to the individual level of fitness of the participants | 9 weeks |  | Not increase significantly |
| (Gmiat et al.,2017) | Untrained healthy woman | HICT | 10 training movements | 10s of transition time, repeated 3 times with 2-minute breaks in-between circuits | Make as many repetitions as possible in time | Each performed for 30s,7min | Before, 1 and 24 h after training | No significant changes |
| (Ozbay et al.,2020) | Healthy males | Aerobic exercise | Winter aerobic training indoor (21°C–25°C) long-term aerobic running exercises | 4 days/week | 65%–70% HR(50%–55% VO2max) | 40 min,18 weeks | Before the 18-week training period,  immediately after the first training,   24 h after the 18‑week training period | Significantly increased* after the first training; significantly decreased* after the 18 week training |
|  |  |  |  |  |  |  |  |  |
|  |  |  |  |  |  |  |  |  |
|  |  |  | Winter aerobic training outdoor (−5°C–5°C) long-term aerobic running exercises |  |  |  |  | Significantly increased * after the first training; unchanged after the 18‑week |
|  |  |  |  |  |  |  |  |  |
|  |  |  |  |  |  |  |  |  |
| Table settings in accordance with the FITT principles, including: frequency, intensity, time, type.  HR:heart rate;HIIE:High intensity interval exercise;CME:Continuous moderate-intensity exercise;HICT:High-intensity circuit training;According to the article,effective irisin was labeled with *,*P<0.05,**P<0.01 | | | | | | | | |
